# Effects of biochar pyrolysis temperature on thermal properties of polyethylene glycol/biochar composites as shape-stable biocomposite phase change materials

**DOI:** 10.1039/d1ra09167k

**Published:** 2022-03-28

**Authors:** Shiwang Liu, Shigui Peng, Bingbing Zhang, Bin Xue, Zhao Yang, Sheng Wang, Guomin Xu

**Affiliations:** Department of Polymer Material and Engineering, College of Materials and Metallurgy, Guizhou University Guiyang 550025 China; National Engineering Research Center for Compounding and Modification of Polymer Materials Guiyang 550014 China 1191117410@qq.com 410034801@qq.com

## Abstract

The characteristics of biochar are of great significance to its application in the field of phase change energy storage. The objective of this research was to explore the effects of pyrolysis temperature on the characteristics of a biochar matrix and further on the heat energy storage properties of the promising green biochar-supported shape-stable biocomposite PCMs (ss-BCPCMs). Corn straw biochars (CSBCs) obtained under different pyrolysis conditions were loaded with polyethylene glycol (PEG) by an ultrasound-assisted vacuum impregnation method. The micro-morphology, specific surface area, pore structure and surface properties of biochar have been characterized and analyzed by scanning electron microscopy (SEM), Brunauer–Emmett–Teller (BET) method and Fourier transform infrared spectroscopy (FTIR). The thermal properties (chemical stability, latent heat storage, thermal conductivity, thermal stability, and thermal insulation) of PEG/CSBC composites have been characterized by FTIR, differential scanning calorimetry (DSC), thermogravimetric analysis (TGA) and laser flash analysis (LFA). The study revealed that both pore structure and surface activity of biochar are key factors affecting the energy storage performance of biochar-based ss-BCPCMs. The obtained PEG/CSBC composite showed a high latent heat storage up to 100.2 J g^−1^, good shape stability and leakage resistance, suggesting its high thermal storage stability that is beneficial for thermal energy storage applications. In addition, its excellent photothermal conversion efficiency (68.95%) provides application potential in photothermal energy storage.

## Abstract 2

The micropore and mesopore of biochar and the interaction between PEG and biochar surface effectively prevented the leakage of PEG and affected the crystallization and adsorption properties of PEG and the heat storage of composite PCMs.

## Graphical abstract

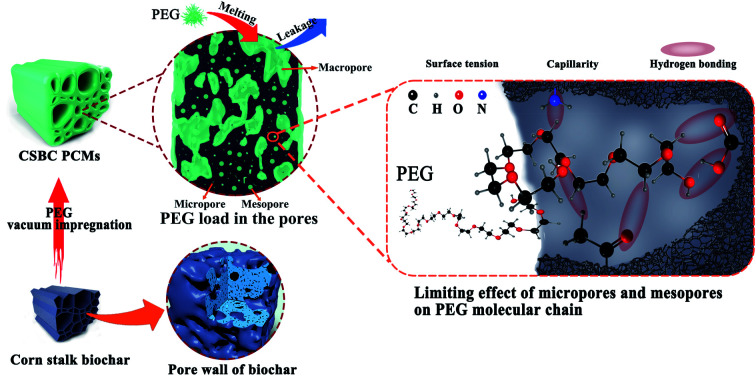

## 1. Introduction

Nowadays the problem of energy shortage is becoming increasingly prominent due to the rapid development of the economy and society. Therefore, developing new energy conversion and storage methods has become a focus of attention. Phase change materials (PCMs), have been widely studied in recent years due to their high storage density, good chemical stability, and capability to store and release thermal energy at a nearly constant temperature, and have been regarded as an excellent approach to alleviate the energy crisis and improve energy storage efficiency.^1,2^

According to the types of phase change, PCMs are generally divided into gas–liquid PCMs, solid–gas PCMs, solid–liquid PCMs, and solid–solid PCMs. And thereinto, solid–liquid PCMs are the most commonly used latent heat storage materials owing to their high latent heat of phase change, stable performance, and easy-to-realize industrial application.^3^ However, the problem of liquid leakage during phase transition remains a major obstacle to their practical application and development.^4,5^ Form-stable (or shape-stable) phase change materials (FSPCM) composites, which could resist leakage by encapsulating the solid–liquid PCMs in an inorganic or organic matrix, are considered as an effective solution to this problem.^5^ Many supporting materials have been applied in FSPCM including mesoporous silica,^6^ carbon nanotubes,^7^ montmorillonite nanosheets,^8^ graphene,^9^*etc.* Recently, biochar (BC) has been widely studied for their affluent raw material, low cost, large surface area, porous structure and thermal conductivity.^10–12^ The study of Jeon *et al.*^10^ indicated that the FSPCC prepared by coconut oil-impregnated biochar showed a high latent heat storage capacity and thermal insulation performance, and the maximum latent heat storage capacity and the thermal conductivity at the maximum were respectively 74.6 J g^−1^ and 0.030 W (m^−1^ K^−1^). Moreover, they also found that the FSPCC prepared by different BC showed disparate latent heat storage capacity because of the differences in physical structure. Differently, Wan *et al.*^13^ found that the FSPCC based on pinecone biochar and palmitic acid not only showed well thermal stability and relatively higher latent heat storage capacity of 84.74 J g^−1^ but also showed a comparatively well thermal conductivity of about 0.3926 W (m^−1^ K^−1^). The recent study reported by Kim *et al.*^14^ showed that the BC prepared by different biomass sources showed diverse structural properties, leading to the capacity of loading/energy storage for organic phase change material shows different greatly. They pointed out that the surface functionality, structural characteristics, type of biomaterials, intermolecular interaction between PCMs and biochar as well as pyrolysis temperature play important roles in determining the thermal properties of the as-prepared FSPCC. Based on the above analysis, it is seen that much factors affect the ultimate heat storage performance of the FSPCC, depending on specific biomass feedstock, processing technology, and PCMs.

In addition, the thermal storage technology based on PCMs has attracted extensive attention in recent years, and the thermal storage performance of PCMs mainly depends on its thermal conductivity and photothermal conversion performance. It is found that addition of carbon nanomaterials such as graphite nano-platelets (GNPs), carbon nanotubes, and graphene nano-platelets into a PCM could accelerate its thermal conduction and photothermal conversion, thus greatly improving its solar thermal energy collection and storage. However, up to now, there is still few studies concerned on both the heat storage and photothermal conversion performance of biochar-based PCMs. In fact, the relationship between processing, structure of biochar and the ultimate photothermal conversion performance of PCMs composites was still unclear. Therefore, further studies were still needed.

In this study, corn straw was selected as raw materials to prepared biochar and polyethylene glycol (PEG) was chosen as PCMs to prepare shape-stable composite. Effect of pyrolysis temperatures on the microstructure of corn straw biochar (CSBC) was carefully studied. Simultaneously, thermal properties of different PEG/CSBS composites were systematically studied, and our effort was focused on the relationship between processing, structure of biochar and the ultimate photothermal conversion performance of PEG/CSBS composites. It is believed that this study might offer a novel approach to design and prepare high-performance PCMs for energy storage industry.

## 2. Material and methods

### 2.1. Materials

The raw materials used to prepare the samples in this research were corn straw and polyethylene glycol (PEG). The corn straw was obtained from Guiyang, Guizhou Province, China. Polyethylene glycol was purchased from Shanghai Alighting Biochemical Technology Co., Ltd. Its average molecular weight, bulk density and latent heat are 2000, 1.13 g cm^−3^ and 213.2 J g^−1^, respectively. Its melting point is 49–53 °C. All reagents used in the experiment were of analytical grade without further purification.

### 2.2. Preparation of corn straw biochar and PEG/CSBC

**Fig. 1:**
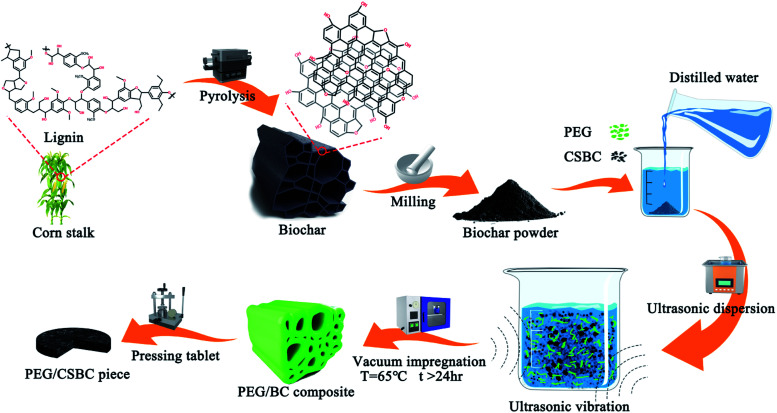
Schematic diagram of the preparation process for CSBC and PEG/CSBC composites.

The schematic diagram of the preparation process was shown in Fig. 1. The dried corn straw was crushed and placed on a quartz ship. Then, the protective gas (nitrogen) with a purity of 99.99% was fed into the tube furnace at a flow rate of 60 mL min^−1^. At a slow heating rate of 10 °C min^−1^, the biochar was heated to different temperatures and kept for 2.5 hours to obtain pyrolyzed corn straw biochar (CSBC). Finally, the CSBC was ground into powder, sieved, and used as the next step for testing. To study the different structures of CSBC, corn straw was pyrolyzed at 400 °C, 500 °C, 600 °C and 800 °C by the above procedure, and named BC400, BC500, BC600, and BC800, respectively.

The vacuum impregnation method was adopted to fabricate a novel kind of shape-stable biocomposite PCMs (ss-BCPCMs) *via* impregnating PEG into the porous structure of CSBC. Firstly, each biochar above (0.5 g) was added into the beaker containing distilled water (25 mL), and then PEG (0.5 g, 50 wt%) was added in. The precursor was dispersed for 90 min by the ultrasonic instrument at room temperature and then dried in a vacuum oven for 24 h at 65 °C. Therewith, the granular sample was obtained when the precursor cooling to room temperature. The obtained composites were called PCS400, PCS500, PCS600, and PCS800, respectively. For convenience of characterization, all samples were made into tablets with 2 mm thickness and 12.7 mm diameter by a tablet press.

### 2.3. Characterization

The microstructure of biochar and PEG/CSBC composites at room temperature was observed by scanning electron microscope (SEM: Quanta FEG 250, American FEI) with an accelerating voltage of 10 kV and a resolution of 2 nm.

Specific surface area and pore structure properties of CSBC were measured by the automatic specific surface area and porosity analyzer (Quantachrome, NOVA-1000e, USA). The specific surface area, micropore surface area, and pore size distribution were measured by Brunauer–Emmett–Teller (BET) method, *t*-plot method, and density functional theory (DFT) method, respectively. Total pore volume for pores with a diameter less than 163.99 nm at *P*/*P*_0_ = 0.988285 was calculated.

Fourier transform infrared spectroscopy (FTIR: Nicolet NEXUS670, USA) was used to monitor the changes of chemical groups on surface of all samples in solid state in the range of 400–4000 cm^−1^. The PEG/CSB composites were mixed with potassium bromide (KBr) and pressed into disks by powder molding for test.

The melting point, freezing temperature, and latent heat capacity of PEG/CSBC composites were measured by differential scanning calorimetry (DSC: TA Q10, America). All samples were heated and cooled at a rate of 5 °C min^−1^ in nitrogen atmosphere in the range of 0–90 °C and 90–0 °C, respectively. The thermal cycle stability of the sample was tested under the above-mentioned rising and cooling procedure, and the test times was 100 cycles.

The thermogravimetric analyzer (TGA: TA Q50, America) was used to analyze the PEG/CSBC composites which were heated to 600 °C at a heating rate of 10 °C min^−1^ under nitrogen atmosphere.

The PEG/CSBC samples were heated to 70 °C, which was higher than the melting temperature of the pure PEG, in the constant temperature oven. The leakage resistance and shape stability of PEG/CSBC composites was recorded by a digital camera.

The crystalline phases of PEG and PEG/CSBC composites were measured by the X-ray diffractometer (XRD, X'Pert PRO). The rate of scanning and range was 10° min^−1^ and 10–80°, respectively.

The thermal conductivity of PEG/CSBC samples was measured at 25 °C by the laser flash method using LFA 467 (NETZSCH) instrument.

The photothermal conversion test was carried out by using an infrared radiation lamp. To maintain the light intensity at 100 mW cm^−2^ during the whole process, a radiometer was employed. The infrared temperature probe was used to record the temperature change at a recording interval of 1 s.

## 3. Results and discussion

### 3.1. Microstructure of the prepared CSBC

**Fig. 2:**
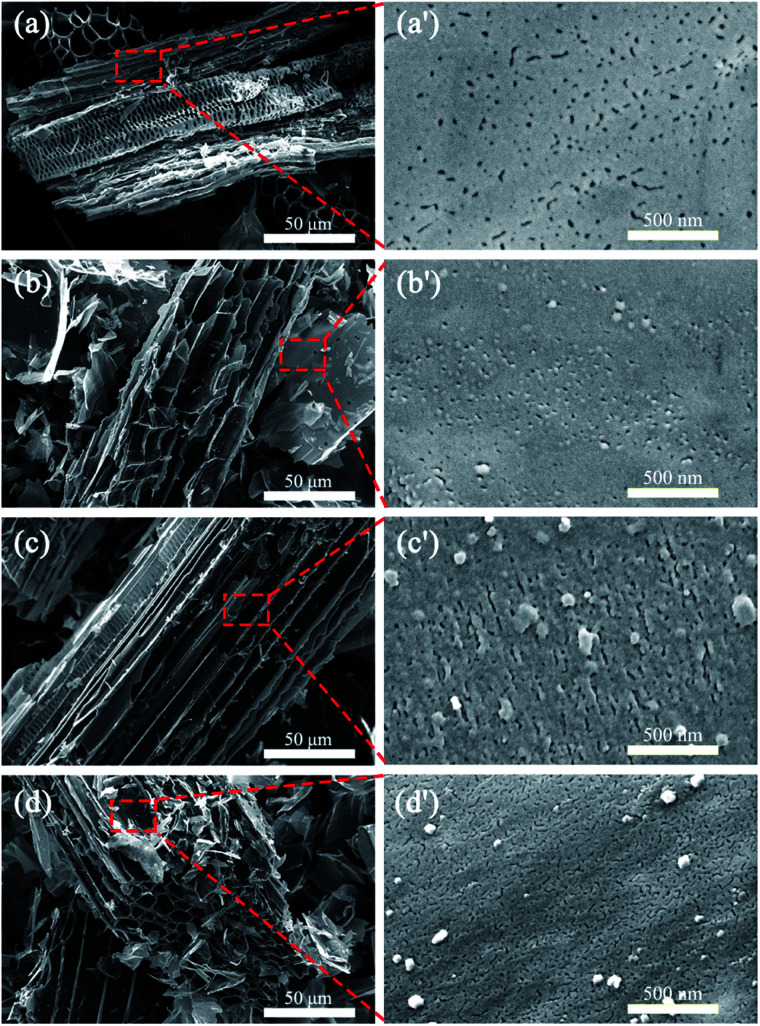
SEM images of (a and a′) BC400, (b and b′) BC500, (c and c′) BC600, and (d and d′) BC800.

Fig. 2 shows the SEM photographs of the CSBCs prepared under different pyrolysis temperatures. It is seen from Fig. 2(a)–(d) that all prepared CSBC showed honeycomb porous structures, with similar trenches and tunnels, but with different pore feature on the wall of these trenches and tunnels as shown in Fig. 2(a′)–(d′). With pyrolysis temperature rose, the pore density and the amount of interconnected pores increased (Fig. 2(a′)–(d′)), the specific surface area grew from 5.382 m^2^ g^−1^ to a maximum of 354.183 m^2^ g^−1^ and the total pore volume grew from 0.0073 cm^3^ g^−1^ to a maximum of 0.1654 cm^3^ g^−1^ (see in Table 1). The results confirm that pyrolysis temperature is a dominant factor affecting the microstructure of biochar, in consistent with other reports.^15–17^ Based on the SEM observations, biochar prepared from rice straw had an outer surface, the external surface of all trenches and tunnels, and an inner surface, the internal pore walls of pores of all sizes. These trenches and tunnels are channels for adsorbates entering the porous system and immobilized on the inner surface of biochar.^18^

**Table 1:** Textural properties of biochars

	BC400	BC500	BC600	BC800
QSDFT average pore diameter (nm)	1.096	0.614	1.007	0.614
Surface area (m^2^ g^−1^)	5.844	11.862	24.397	354.183
Micropore surface area (m^2^ g^−1^)	5.382	6.504	15.090	314.700
Total pore volume^a^ (cm^3^ g^−1^)	0.0073	0.0163	0.0226	0.1654
Micropore volume^b^ (cm^3^ g^−1^)	0.002283 (0.0021)	0.009812 (0.0065)	0.01529 (0.0088)	0.1583 (0.1278)
Mesopore volume^c^ (cm^3^ g^−1^)	0.000722	0.000266	0.00184	0.000174

Proportion in total pore volume^d^ (%)				
Micropore	31.27	60.20	67.65	95.71
Mesoporous	9.89	1.63	8.14	0.11
Macropore	58.84	38.17	24.20	4.19

a Note: total pore volume for pores with diameter less than 158.57 nm at *P*/*P*_0_ = 0.987882.

b DR-method micropore volume and T-method micropore volume in parentheses.

c Statistical QSDFT volume histogram with pore width of 2.05–50 nm.

d The volume of micropores, mesopores and macropores mentioned above divided by the total pore volume, respectively.

**Fig. 3:**
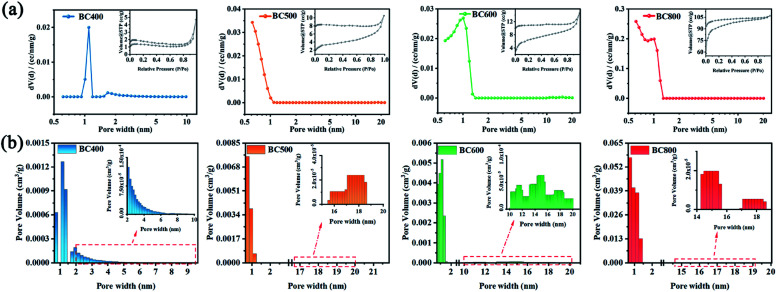
Pore characteristics of biochar prepared: (a) N_2_ adsorption–desorption isotherm and pore size distribution curve, (b) pore size distribution histogram.

To further understand the influence of pyrolysis temperatures on pore structure of CSBCs, the nitrogen adsorption–desorption method was employed. It is seen all prepared CSBCs showed type II sorption–desorption isotherms (Fig. 3(a)), with the type H3 hysteresis loop appearing on. The typical open hysteresis loop refers very narrow slit pores.^19^ With the increasing of pyrolysis temperature of the CSBC, the sorption–desorption isotherm curve presented gradually aggrandizing, implying an increasing porosity.

The specific structural parameters of the prepared CSBC were summarized in Table 1. As expected, the total pore volume and surface area of the prepared CSBC was enhanced by promoting the pyrolysis temperature. As shown in Table 1, the total pore volume and the surface area of BC800 were 0.1654 cm^3^ g^−1^ and 354.183 m^2^ g^−1^ respectively, which was about 22 times and 60 times that of BC400. What was noteworthy was that the pore distribution of the CSBC prepared at different pyrolysis temperatures showed a difference. It was seen that the micropore volume of BC400, BC500, BC600, BC800 were respectively 0.0021 cm^3^ g^−1^, 0.0065 cm^3^ g^−1^, 0.0088 cm^3^ g^−1^, 0.1278 cm^3^ g^−1^ (according to Fig. 3(b)) and the proportion of micropore volume to total pore volume were 31.27%, 60.20%, 67.65% and 95.71%, respectively, while the proportion of macropores decreased from 58.48% to 4.19%. The increasing of pyrolysis temperature of biochar leads to a more microporous, less microporous microstructure. This is because as the pyrolysis temperature (800 °C) increased, more volatile substances, as well as carbon dioxide, water and methane were released, and the carbon structure collapsed at a higher pyrolysis temperature, resulting in more micropores.^20,21^ Meanwhile, the mesoporous proportion of BC400 and BC600 are closer and much higher than that of other two. Unexpectedly the tendency of the average pore diameter at biochars were not in according with the former explanation completely. BC400 showed the largest average pore diameter was about 1.096 nm, and that of BC600, BC500, BC800 were 1.007, 0.614, 0.614 respectively. Whatever, the fact could be confirmed that the pyrolysis temperature of biochar plays a significant role on the pore size of biochar.^22^

**Fig. 4:**
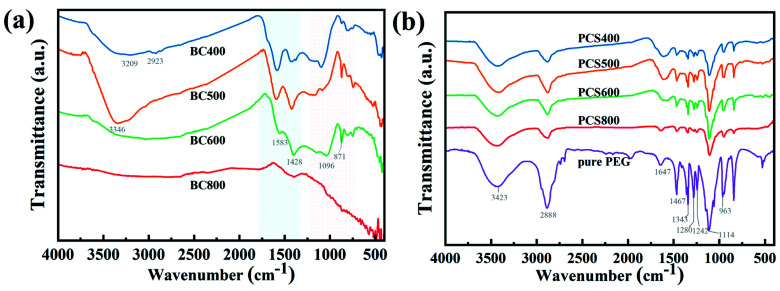
FT-IR spectrum of biochar (a) and as-prepared composite PCMs (b).

To determine the surface functionality of the prepared CSBC, FTIR was carried out and the result was shown in Fig. 4(a). The broad absorption peaks at approximately 3309–3446 cm^−1^ can be ascribed to the stretching vibration of –OH,^23,24^ the peaks at approximately 1583 cm^−1^ and 1428 cm^−1^ were attributed to C=C and C=O stretching respectively,^24^ and the weak peaks at 1096 cm^−1^ and 871 cm^−1^ were attributed to C–O and C–H stretching vibration. Obviously, all the prepared CSBC showed abundant oxygen-containing functional groups on the surface except for BC800. It was reported that the nitrogen-and oxygen-containing functional groups on the surface of biochar improved the stability and physical adsorption capacity of guest materials through intermolecular interaction such as hydrogen bonding.^25^

### 3.2. Thermal energy storage and stability property of PEG/CSBC composites

**Fig. 5:**
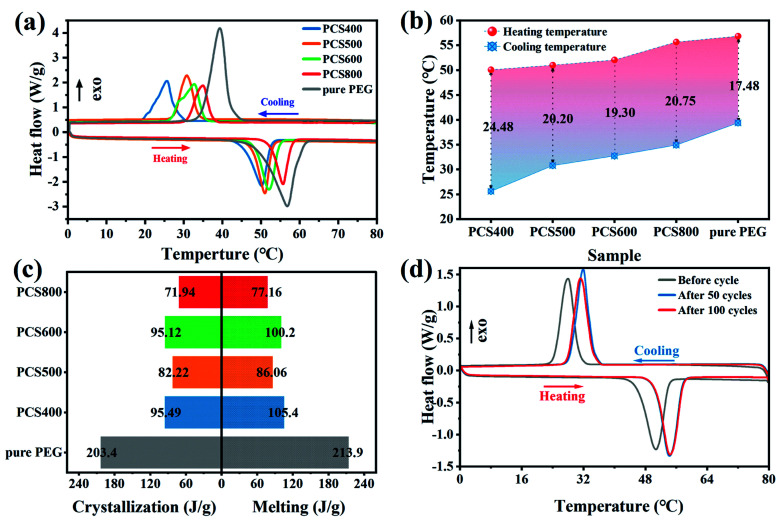
Heat storage performance of the as-prepared of PEG/CSBC and PEG: (a) DSC curve, (b) phase-change temperature, (c) phase transition enthalpy, and (d) thermal cycle curve.

The phase change behaviors of pristine PEG and PEG/BC composites, including the phase change temperatures and latent heats, were evaluated using the DSC technique. The DSC curves of pristine PEG and PEG/CSBC composites was shown in Fig. 5(a) and the calculated test data were summarized in Table 2, Fig. 5(b) and (c). As shown in Fig. 5(a), the melting and solidification temperature as well as the latent heat of pristine PEG were 56.84 °C, 39.36 °C and 213.9 J g^−1^, 203.4 J g^−1^, respectively, but those of PEG/BC composites have gotten lower. The phase-change temperature of composite with lower temperature pyrolyzed CSBC has fallen further owing to the strong interactions between the PEG and supporting material.^26^ Such strong interaction force could cause a constraint effect limiting the normal motion of PCM during phase transition.^27,28^ Meanwhile, the prepared biochar shows a microporous structure (see Fig. 3(a)), and the decrease of crystallization temperature of composite PCMs was observed in Fig. 5(b). The micro structure effect of biochar was explained. More specifically, when the crystallization was limited to the micro–nano region, a typical phenomenon was that the crystallization temperature was reduced.^29,30^ In addition, PCS800 showed a lower enthalpy. The reason may be the difference of surface properties and pore structure of PEG in biochars. Usually, the composite PCMs with higher PCMs adsorption capacity exhibited higher enthalpy. Significantly, PCS400 and PCS600 have showed relatively good thermal energy storage performance with the latent heat during melting/solidification of 105.4/95.12 J g^−1^ and 100.2/95.49 J g^−1^ respectively, being competitive with recently reported FSPCMs (Table 4) such as coconut shell activated carbon/polyethylene glycol (90.2 J g^−1^),^31^ almond shells biochar/polyethylene glycol (82.73 J g^−1^)^11^ and potato activated carbon/polyethylene glycol (91.80 J g^−1^).^32^

**Table 2:** Thermal and photothermal conversion properties of the PEG and the ss-BCPCM

Sample	*T*_m_ (°C)	Δ*H*_m_ (J g^−1^)	*T*_c_ (°C)	Δ*H*_c_ (J g^−1^)	Light intensity *P* (mW cm^−2^)	Phase transition time Δ*t* (s)	Photothermal conversion efficiency *η* (%)
Pure PEG	56.84	213.9	39.36	203.4	100	946	44.63
PCS400	50.09	105.4	25.61	95.49	100	412	49.25
PCS500	51.00	86.06	30.80	82.22	100	374	50.54
PCS600	52.04	100.2	32.74	95.12	100	299	68.95
PCS800	55.68	77.16	34.93	71.94	100	311	55.55

The thermal stability and reliability of PCS600 was furtherly confirmed by the freeze–thaw cycling test in 100 times. The results shown in Fig. 5(d) indicated that all thermal cycle curves had similar characteristic peaks, with a high enthalpy of fusion (98.93 J g^−1^) after 100 cycles retaining 98.7% capacity of the initial, proving the excellent cycle stability of PCS600. The thermal parameters of freeze–thaw cycling were shown in Table 3.

**Table 3:** Thermal properties of PCS600 after cold and thermal cycling

Number of cycles	*T*_m_ (°C)	Δ*H*_m_ (J g^−1^)	*T*_c_ (°C)	Δ*H*_c_ (J g^−1^)
Before cycle	50.72	100.2	28.02	95.12
After 50 cycles	54.41	98.31	34.40	96.68
After 100 cycles	54.21	98.93	34.51	97.18

**Table 4:** Comparison of thermal properties of different PCMs in literature

Sample name	Loading material	Latent heat Δ*H*_m_ (J g^−1^)	Concentration (wt%)	Ref.
PEG/PAN	Polyaniline	70	50	Sarier, *et al.*^35^
PEG/ASB	Almond shells biochar	82.73	60	Chen, *et al.*^11^
PEG/AC	Coconut shell activated carbon	90.2	—	Feng, *et al.*^31^
PEG/AC	Potato activated carbon	91.80	50	Tan, *et al.*^32^
PA^a^/PB	Pine cone biochar	84.74	60	Wan, *et al.*^13^
PA–LA^b^/CAR	Carbonized waste rice	135.4	79.7	Zhang, *et al.*^36^
CA–PA–SA^c^/AC	Activated carbon	67.15	62.92	Yuan, *et al.*^37^
PW^d^/R	Rice husk biochar	92.13	64.77	Jeon, *et al.*^12^
PEG/CSBC	Corn straw biochar	105.4	50	Present study

a Palmitic acid.

b Palmitic acid–lauric acid.

c Decanoic–palmitate–stearic acid.

d Palm wax.

**Fig. 6:**
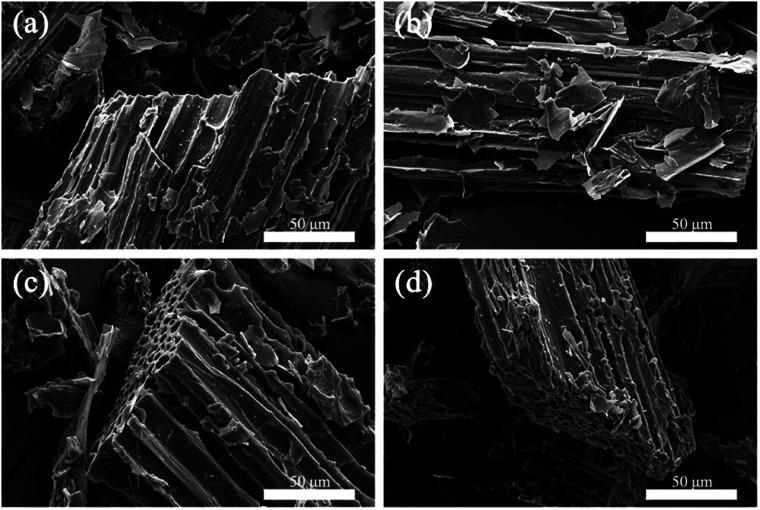
Microscopic surface SEM images: (a) PCS400, (b) PCS500, (c) PCS600, and (d) PCS800.

**Fig. 7:**
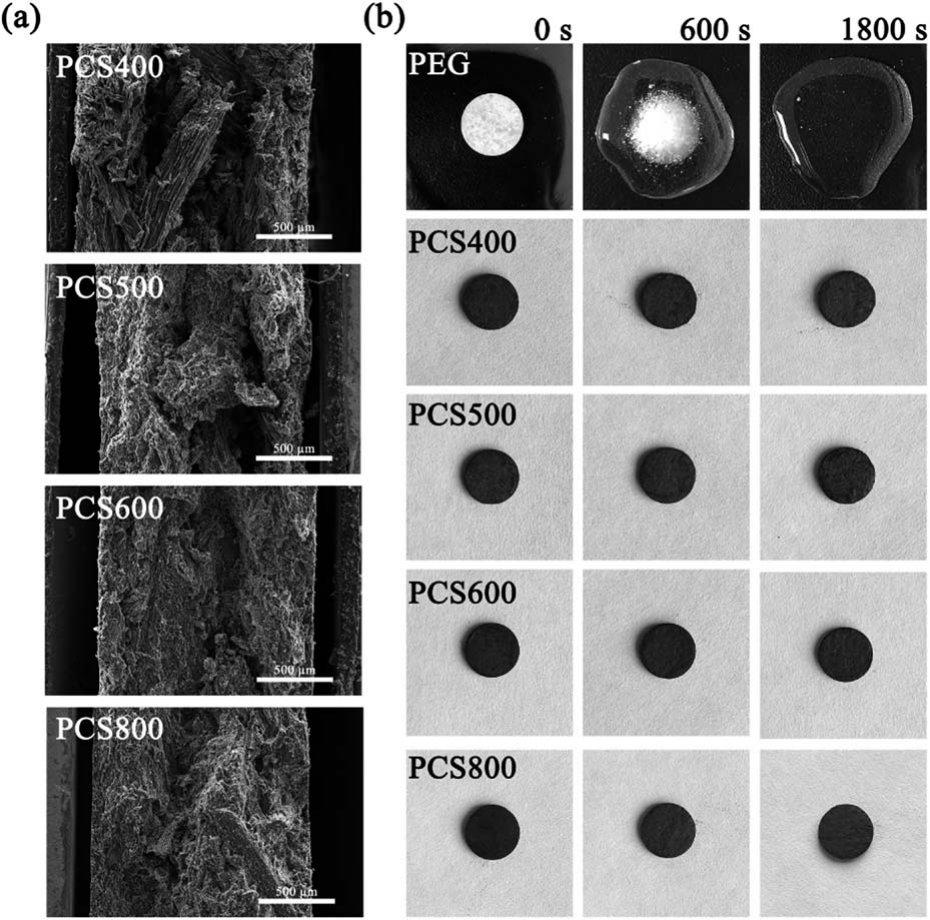
Cross-sectional SEM images of as-prepared composite PCMs (a) and leakage test (b).

The apparent morphologies of PEG/CSBC composites and their cross-sections were obtained by SEM as images shown in Fig. 6 and 7(a), while the photographs of the PCMs tablets at 70 °C in 0, 600, 1800 s were recorded for leakage test observation. As shown in Fig. 6 and 7(a), the surface of composites remained smooth, and PEG was totally introduced into the surface and pore of biochars. With theoretical 50 wt% PEG content, the macropores on biochars have't been filled fully still, indicating the strong adsorption capacity of the porous material for PEG. In Fig. 7(b), pure PEG gradually melted as test time goes on and completely melted at 1800 s. On the contrary, the as-prepared PEG/CSBC composites have retained a stable form without any liquid leakage during the process, due to the capillarity, strong surface tension, and hydrogen bond interaction of microstructure of biochars towards PEG.^33,34^ Eventhough a small amount of fluid spilled from the microstructure, it would be restrained in granular interspace of the pressed PCMs tablets. Therefore, the CSBC can be confirmed as an effectively support for PCMs organic molecules.

The FTIR spectra of the PEG/CSBC were shown in Fig. 4(b) for further investigation of their chemical stability. It showed that the functional group (–OH, C–O(H)) was observed on the characteristic curve of PEG. There was a strong –CH_2_ stretching absorption peak at 2884 cm^−1^ and 961 cm^−1^,^38^ a hydroxyl (–OH) stretching absorption peak at 3430 cm^−1^, a C=O double bond stretching absorption peak at 1647 cm^−1^, and a C–O single bond stretching oscillation peak at 1115 cm^−1^. The peak of the C–H bond was found at 1462 cm^−1^ and 1338 cm^−1^, while the peak of the hydroxyl group was found at 1276 cm^−1^ and 1242 cm^−1^.^39^ No additional characteristic peaks with the exception of those on PEG and CSBC have been observed on PEG/CSBC, that means no chemical reactions have occurred during the impregnation process, ensuring the phase transition of composite PCMs for heat storage,^40^ proving good chemical stability of the composite PCMs. Moreover, it was worth noting that the peak near 1647 cm^−1^ in pure PEG was the absorption peak of C=O stretching vibration, which was also observed in composite PCMs. Also it can be seen that the wave number of corresponding absorption peak on PCS400-600 decreased slightly, indicating an interaction between PEG and biochars.

**Fig. 8:**
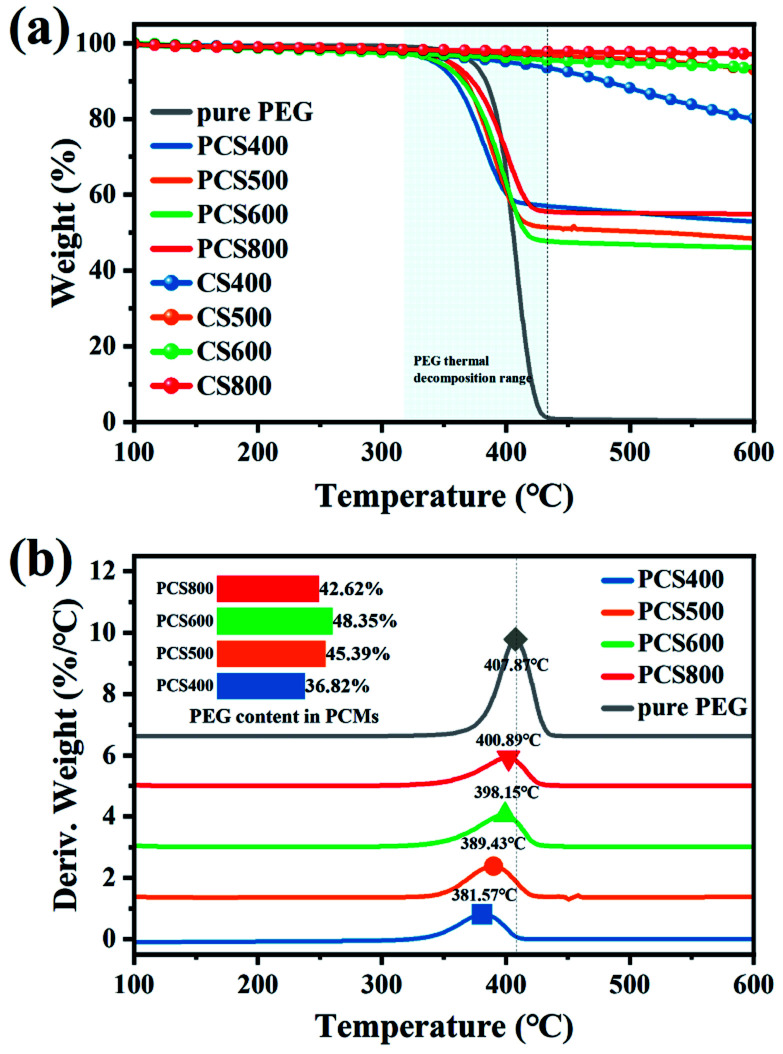
Stability of the as-prepared samples: (a) TGA thermograms and (b) DTG curves and PEG loading.

According to previous studies, the adsorption performance of the carrier to PCM has an important influence on the latent heat of ss-BCPCM. To deeply understand the effect of biochars microstructure on the adsorptivity and the thermal stability of composite PCMs, TG curves of pure PEG, CSBCs and PEG/CSBC composites with constant designed loading ratio was studied. As can be seen from Fig. 8(a), the TG curve of pure PEG started to decline from about 300 °C and ended up with 450 °C, and the weight remains unchanged thereafter. The maximum weight loss rate of pure PEG and PCS400-800 occurred at 407.87 °C and 381.57 °C, 389.43 °C, 398.15 °C, 400.89 °C (Fig. 8(a) and (b)), respectively, implying the higher pyrolysis temperature of biochar, the more abundant micropores of supports, the better thermal stability of composites. Comparison to the thermal stability of pure PEG, that of PSC400 has been significantly weakened by the exist of BC400, but barely changed under 100 °C. As for CSBCs, slight weight loss can be observed except for BC400, because it has kept losing its graphitic carbon after 450 °C. It has been reported that the pore volume determines the overall adsorption rate of PCMs, which determines the loading capacity.^28^ According to the data in Table 1, the total pore volume of biochar increased with the increase of pyrolysis temperature. Significantly, as shown in the embedded figure in Fig. 8(b), the practical loading ratio of PEG in PEG/CSBC, equating with the weight loss rate of PEG in PCS400, PCS500, PCS600 and PCS800 exclusive of the loss of CSBCs, were 36.82%, 45.39%, 48.35% and 42.62%, respectively. It showed that the pore volume of biochar was not the only factor affecting the overall loading rate of biochar to PEG. Its surface activity also played a significant role in the loading capacity of PCMs. The pore structure and surface properties were reciprocal to determine thermal performance of biochar-based PCMs. Although porosity of PCS800 was the highest, its adsorption capacity was low because of the relatively inert surface, resulting in a low latent heat (see Table 2). These results directly revealed the effect of biochars microstructure and surface activity on their loading capacity towards PEG. In addition, the trend of pyrolysis peaks on composite PCMs in Fig. 8(b) coincides with the analysis based on FT-IR results above.

**Fig. 9:**
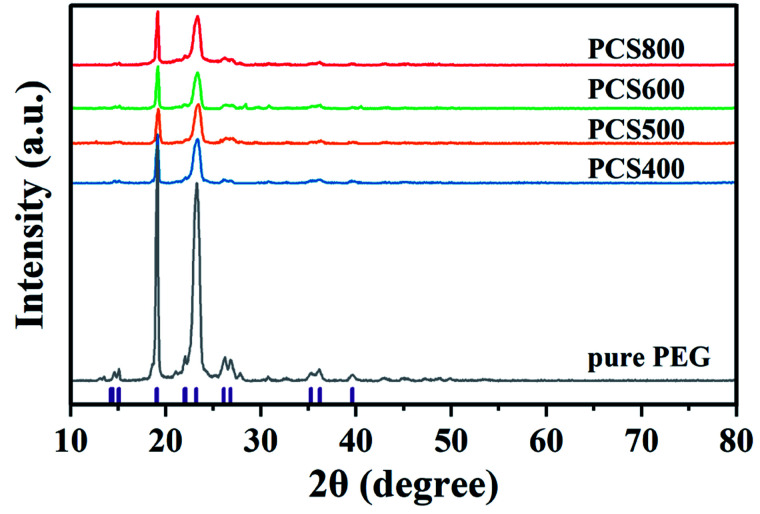
XRD spectra of the as-prepared composite PCMs.

To further explore the changes in the crystallization properties of the PEG/CSBC composites, we evaluated the grain size and crystallinity of the composites according to the XRD curves shown in Fig. 9, and the statistical results were shown in Table 5. The XRD results of pure PEG showed that there were four obvious peaks at the 2*θ* of 19.1°, 23.2°, 26.1° and 26.8° representing the regular crystal structure of PEG.^41^ And it can be seen from the XRD curves of PEG/CSBC that the characteristic peaks of PEG were almost shown as well but with much weaker intensity. This may be due to the part of the PEG was confined in the pores, which hindered their normal crystallization and molecular arrangement, resulting in the change of the intensity of the diffraction peaks. Moreover, the diffraction angle 2*θ* of the composite PCMs shifts slightly at 19.1° and 23.2°, which indicates that there is an interaction between PEG and biochar, consistent with the results of FT-IR and DTA analysis. The average grain thickness (*D*) in PEG/CSBC composites was used to estimate the effect of CSBC microstructure on the crystallization properties of PEG, and calculated by the following formula (1):^42^1
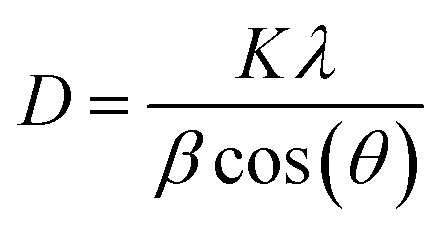
where *K* is a constant, *λ* is the X-ray wave length, *β* was full width at half maximum (FWHM), and *θ* is the Bragg reflection angle.

**Table 5:** The crystal size and crystallinity of composites

	Pure PEG	PCS400	PCS500	PCS600	PCS800
Crystal size (nm)	14.573	14.781	9.192	12.734	9.014
Relative crystallinity (%)	100	21.49	19.38	18.16	31.06

As shown in Table 5, the average crystal size of the composites estimated according to eqn (1) was in the range of 9.014–14.781 nm. The crystal size of PEG in PEG/CSBC composites shows high relevancy of the average pore width, pore distribution of CSBCs. In particular, the crystal size of PEG in PCS400 was very close to that of pure PEG, while the smallest was gianed in PCS800. It could be a mass of micropore of BC800 greatly restricted the growth of PEG spherulites. Therefore, the limiting effect of pore structure could be a reason to explain why PCS800 showed a low latent heat. Furthermore, the relative crystallinity of samples was calculated according to the proportion of the crystallization peak total area of each sample to the pure PEG. The difference of PEG crystallinity in composites may be attributed to the different pore structures of the supporting materials.^43^ In PCS400, PCS500 and PCS600 composites, there was an interaction between PEG and porous biochar. The micropore proportion of biochar increased gradually, and most PEG were restricted, which was the reason for the gradual decrease of PEG crystallinity in composites. But unexpectedly, the crystallinity of PEG in PCS800 was higher than that of other samples. The pores of CS400, CS500 and CS600 were composed of micropores, mesopores and macropores (shown in Table 1). Therefore, PEG was limited not only in micropores, but also in mesoporous or macropores, and there was also the influence of surface groups. By contrast, the PEG molecule in CS800 was limited only by abundant micropores. It could be the main reason for the crystallinity of PEG in PCS800 was relatively high. In general, the crystallization performance of PEG was reduced significantly in the presence of biochars.

**Fig. 10:**
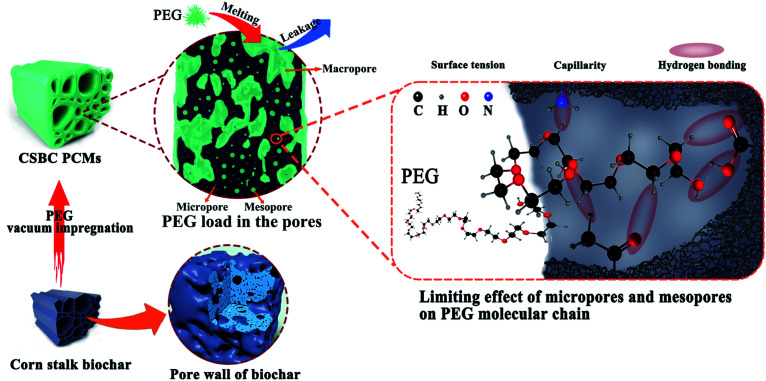
Schematic diagram: the limiting effect of biochar pore structure and its surface groups on PEG molecules.

According to the comprehensive analysis of the above characterization data, it was found that the latent heat of the as-prepared composite PCMs was directly affected by micropores, mesopores structure and surface activity of biochar. Furthermore, it was reported that PCMs capsulated in supporting materials with large pore are prone to leakage in the process of phase transition, which reduces their adsorption to PCMs. But the surface of matrix leading a strong molecular interaction towards PCMs will resist liquid leakage.^44^ The total proportion of micropores and mesopores of the prepared biochar has an important influence on the PEG loading capacity of the composite PCMs (see in Table 1 and Fig. 8(b)). Although the lowest proportion of macropores was found on BC800, it is worth noting that its loading ratio of PEG was low owing to scarce surface binding sites caused by over-pyrolysis process at 800 °C.^45,46^ As a result, the latent heat of PCS800 was low. The effects of surface activity of supporting materials on loading ratio and leakage resistance of PEG/CSBC ss-BCPCMs can be confirmed by FT-IR and DTG analysis as Fig. 4(a) and 8(b) shows. Thus, these results suggest that the porous structure of biochar has a certain adsorption effect of PEG, but does not determine it. And the synergistic effect of pore structure and surface activity can enhance the adsorption performance of biochar for PEG. The influence of pore structure and surface binding sites on the latent heat of composite PCMs was described in Fig. 10.

In addition, the pore size of the carrier not only affects the entrapment efficiency and loading, but also determines the difficulty of crystallization of PCM in the carrier. For example, the degree of freedom in the pore limited the motion of PCMs molecules, which directly limited the reversible crystallization. In contrast, larger pores provided enough space for crystal growth to grow into larger crystals. Meanwhile, the organic phase change materials will not undergo phase transition in the pores below 5 nm, 5–50 nm is a more suitable pore size in order to achieve reversible crystallization effectively.^47^ It was worth noting that there was a significant relationship between the mesoporous proportion of prepared biochar, the latent heat and the average spherulites size of PEG of ss-BCPCMs (see Tables 1, 2 and 5). And because the three-dimensional pore network will restrict the growth of PEG spherulites, the latent heat of the composites was lower than that of the original PEG.^33^ Chiarappa *et al.* established a thermodynamic model to predict that the melting temperature and enthalpy of organic drugs decrease with the decrease of nanocrystal size.^48^ It was found that the influence of the shape and morphology of nanocrystals on the melting temperature and enthalpy decreased with the decrease of grain size. Therefore, it was speculated that the latent heat of the biochar composite PCMs was related to the mesoporous proportion. That was, the higher the mesoporous proportion, the more space for crystal growth, the average grain size of PEG increased, and then the overall latent heat increased.

### 3.3. Thermal conductivity and photothermal properties of PEG/CSBC composites

**Fig. 11:**
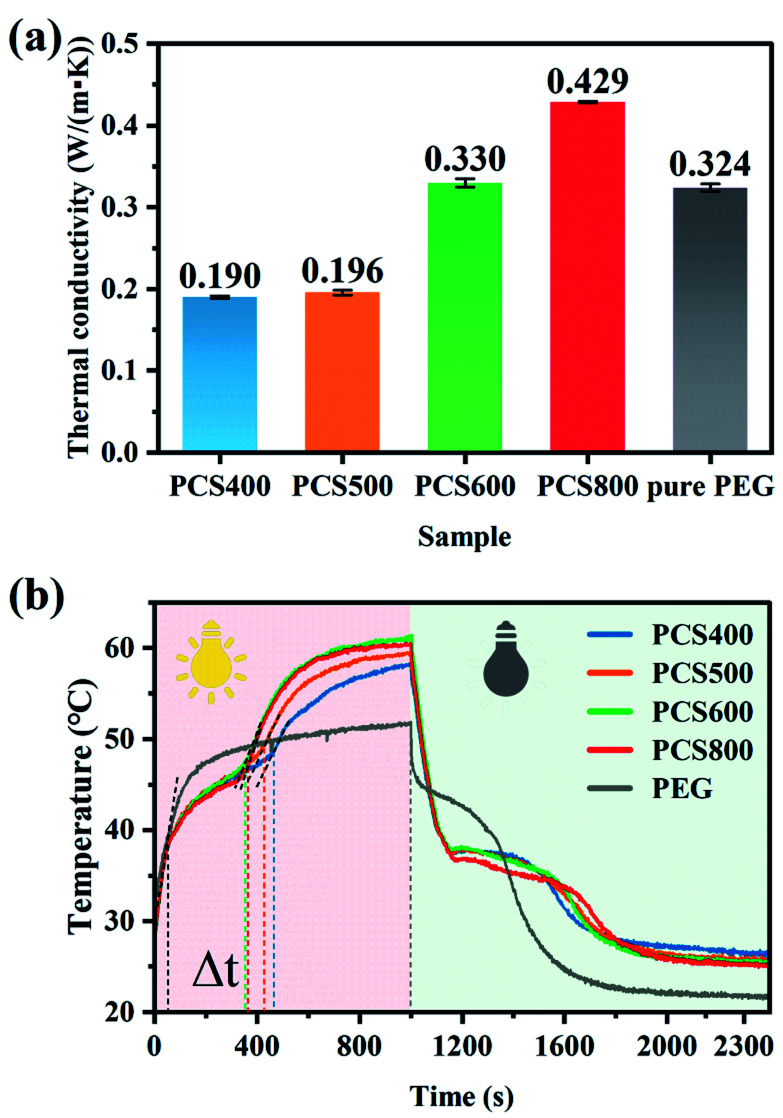
Thermal conductivity at 25 °C (a) and photothermal conversion curves (b) of the prepared materials.

The thermal conductivity (*K*) value of the composite indicates the ability of the material to conduct heat energy.^49^ It provided information about the relationship between heat conduction and thermal storage and can be assessed from the following eqn (2):

2 *K* = *α* × *ρ* × *C*_p_

where *K* is the thermal conductivity, *ρ* is the density, and *C*_p_ is the specific heat of the prepared sample. The thermal conductivity of the composites was in the range of 0.190–0.429 W (m^−1^ K^−1^) as shown in Fig. 11(a). The high-temperature pyrolysis biochar showed a high thermal conductivity after adsorbing PCM. The result was consistent with the finding of Restucia *et al.*,^50^ that the higher pyrolysis temperature of biochars leads to the higher thermal conductivity of the composite PCMs. Although compared with other carbon-base composite PCMs,^51–54^ all PEG/CSBC composites showed relatively low thermal conductivity, PCS400 exhibited a high latent heat of phase transition.

In this work, infrared radiation was used to drive the energy conversion and energy storage of PCMs. Under infrared radiation, the temperature of sample raised. At the same time, time–temperature data were automatically recorded using an infrared probe recorder connected to the computer. The photothermal conversion curve of the composite PCMs was shown in Fig. 11(b). It can be seen that the temperature of the composite PCMs irradiated by infrared light increased rapidly, and it only took about 60 s to rise from room temperature to phase-change temperature. When its temperature reached to phase transition point, the solid to liquid phase-transition began, and a slowly rising platform appeared on the temperature-rising curve afterwards. Since the endothermic phase-transition has consumed part of the infrared heat energy, the temperature-rising on the composite decelerated. Compared with the black-colored PEG/CSBC composite, the white-colored tablets of pure PEG can be observed apparent transmission and refraction of infrared light on it during the experiment, reducing its light absorption. And because of the low light absorption efficiency of pure PEG, the heating platform on it was obviously longer than that of the composites. When the infrared light was turned off after continuous illumination for 1000 s, the temperature of pure PEG (51 °C) kept lower than that of the composites, while the constant temperature platform was also longer than that of the composites. According to photothermal conversion curves of the composites, the lasting time of phase transition (Δ*t*) on all PEG/CSBC samples in the illumination process were close. The phase transition platform of PCS400 was the longest, and that of the PCS600 and PCS800 were close, and both shorter than that of PCS400. The reason was that the high-temperature pyrolysis biochar with high thermal conductivity can quickly transfer heat energy from the upper layer to the lower layer, increasing the temperature of the composite PCMs and shortening the thermal energy storage time, while the poor thermal conductivity of PCS400 caused the inefficient transfer of infrared thermal radiation into PEG molecules in time.

When the infrared heat source was turned off after continuous irradiation for 1000 s, it can be seen that the reverse phase transition platform of PCS800 was the longest. The reason was that the absorbed heat energy efficiently transferred *via* the high thermal conductivity biochar skeleton to more PEG molecules causing the polymer response immediately, that have reduced the loss of thermal energy to the air, so that the longer phase-change platform was shown. In contrast, the cooling platform of pure PEG was the shortest, because only few PEG crystals have dissolved under radiation for its poor light absorption and thermal conductivity. In addition, the photothermal conversion efficiency of PEG and composite PCMs can be calculated based on the following calculation formula (3):^55^3
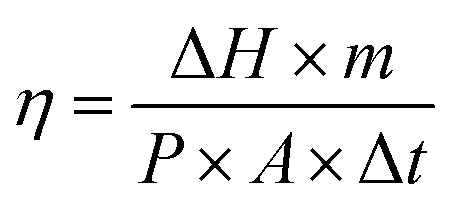
where *m* is the mass of the sample, Δ*H* is the phase transition enthalpy of the sample, *P* is the light intensity, *A* is the irradiated area of the sample, and Δ *t* is the phase transition time of the sample. According to this equation, the maximum photothermal conversion efficiency of PEG and composite PCMs was 44.63% and 68.95%, respectively, and all calculated results were shown in Table 2. The results confirmed that the introduction of CSBC greatly improved the photothermal conversion efficiency of PEG.

## 4. Conclusions

In summary, the “green” composite PCMs with stable shape and excellent thermal stability based on different pyrolysis-temperatures biochars were designed. The effect of the microstructure of biochar on the thermal properties of composite PCMs and further their possibility as green energy storage materials were explored. The results show that the pore size distribution and surface activity of biochar are the main factors in affecting of the energy storage properties of biochar-based PCMs. High pyrolysis-temperature endows biochar with high micropore ratio, narrow pore size distribution (0.614 nm) and low surface activity. Increasing the proportion of micropores and mesopores can increase the PEG loading of biochar, but the pore structure is not the only factor that determines the adsorption properties. The synergistic effect of surface activity and pore structure of biochar can better improve the adsorption performance. In addition, micropores and mesopores have important effects on the crystallization of PEG molecules, and the content of mesopores will greatly affect the latent heat of the composites.

Moreover, in this work, biochar, as a good adsorbent, can make the latent heat of composite phase change materials reach to 71.94–105.4 J g^−1^ in the melting points range of 27.88–52.44 °C. After the freeze–thaw cycling test, the latent heat of PCS600 has reduced merely 1.3% with excellent shape stability and leakage resistance. All prepared samples have good thermal stability, tolerable thermal conductivity, and have enhanced photothermal conversion efficiency by 54.5% higher in comparison with pure PEG. The novel PEG/CSBC biochar-base ss-BCPCMs have potential application prospect in energy storage industry.

## Conflicts of interest

There are no conflicts to declare.

## Supplementary Material
